# Cerebral gray matter volume in patients with chronic migraine: correlations with clinical features

**DOI:** 10.1186/s10194-017-0825-z

**Published:** 2017-12-08

**Authors:** Gianluca Coppola, Barbara Petolicchio, Antonio Di Renzo, Emanuele Tinelli, Cherubino Di Lorenzo, Vincenzo Parisi, Mariano Serrao, Valentina Calistri, Stefano Tardioli, Gaia Cartocci, Anna Ambrosini, Francesca Caramia, Vittorio Di Piero, Francesco Pierelli

**Affiliations:** 1 Research Unit of Neurophysiology of Vision and Neurophthalmology, G.B. Bietti Foundation-IRCCS, Via Livenza 3, 00198 Rome, Italy; 2 Department of Neurology and Psychiatry, Sapienza University of Rome, Rome, Italy; 3 Don Carlo Gnocchi Onlus Foundation, Milan, Italy; 4 Department of Medico-Surgical Sciences and Biotechnologies, Sapienza University of Rome Polo Pontino, Latina, Italy; 5 IRCCS-Neuromed, Pozzilli, IS Italy

**Keywords:** Cerebellum, Temporal pole, Orbitofrontal cortex, Gray matter, Acute medication

## Abstract

**Background:**

To date, few MRI studies have been performed in patients affected by chronic migraine (CM), especially in those without medication overuse. Here, we performed magnetic resonance imaging (MRI) voxel-based morphometry (VBM) analyses to investigate the gray matter (GM) volume of the whole brain in patients affected by CM. Our aim was to investigate whether fluctuations in the GM volumes were related to the clinical features of CM.

**Methods:**

Twenty untreated patients with CM without a past medical history of medication overuse underwent 3-Tesla MRI scans and were compared to a group of 20 healthy controls (HCs). We used SPM12 and the CAT12 toolbox to process the MRI data and to perform VBM analyses of the structural T1-weighted MRI scans. The GM volume of patients was compared to that of HCs with various corrected and uncorrected thresholds. To check for possible correlations, patients’ clinical features and GM maps were regressed.

**Results:**

Initially, we did not find significant differences in the GM volume between patients with CM and HCs (*p* < 0.05 corrected for multiple comparisons). However, using more-liberal uncorrected statistical thresholds, we noted that compared to HCs, patients with CM exhibited clusters of regions with lower GM volumes including the cerebellum, left middle temporal gyrus, left temporal pole/amygdala/hippocampus/pallidum/orbitofrontal cortex, and left occipital areas (Brodmann areas 17/18). The GM volume of the cerebellar hemispheres was negatively correlated with the disease duration and positively correlated with the number of tablets taken per month.

**Conclusion:**

No gross morphometric changes were observed in patients with CM when compared with HCs. However, using more-liberal uncorrected statistical thresholds, we observed that CM is associated with subtle GM volume changes in several brain areas known to be involved in nociception/antinociception, multisensory integration, and analgesic dependence. We speculate that these slight morphometric impairments could lead, at least in a subgroup of patients, to the development and continuation of maladaptive acute medication usage.

**Electronic supplementary material:**

The online version of this article (10.1186/s10194-017-0825-z) contains supplementary material, which is available to authorized users.

## Background

Migraine is a brain disorder that is highly prevalent in the general population and very disabling. The level of disability increases progressively with the attack frequency, reaching its maximum when migraine becomes chronic (CM). A history of frequent migraine attacks and analgesic overuse are the most prominent risk factors for developing CM [1]. However, the neurobiological mechanisms by which some migraineurs develop CM and enter the vicious cycle of medication overuse are still under debate.

During the past decade, a few neuroimaging studies have explored the macrostructural characteristics of the brain in patients with CM, with inconsistent results. One consistent finding though is significant abnormal gray matter (GM) volume in areas ascribable to the processing of pain [2–6] and multisensory integration [5] in patients with CM versus healthy individuals. It should be noted that many of these studies had major sources of bias, namely the inclusion of patients with the following: previous or actual history of medication overuse headache (MOH) [6], concomitant use of preventive medications [3, 6], mixed migraine (with and without aura) [3, 4], and white matter (WM) abnormalities [4]. Hence, studies that avoid such biases are necessary to reveal the mechanisms underlying CM.

Among the various magnetic resonance imaging (MRI) analysis techniques, voxel-based morphometry (VBM) allows for the semi-quantitative estimation of the GM volume of the whole brain [7]. Therefore, the aim of this study was to investigate the brain morphometry in a group of de novo patients diagnosed with CM, i.e. those without a previous history of medication overuse, drug withdrawal, WM abnormalities, and migraine auras, and compare it with the morphometry in healthy controls (HCs). This study also aimed to explore whether there is a relationship between the morphological pattern and the clinical features of CM. Considering the abovementioned studies and our prior ictal/interictal observations in episodic migraine [8], we reasoned that patients with CM would show morphometric changes in brain areas devoted to pain processing and multisensory integration.

## Methods

### Participants

Among the patients who were consecutively admitted to our headache clinics, 20 patients (Table 1) provided informed consent to participate in the present study. Per the International Classification of Headache Disorders, 3rd edition, beta (ICHD-3 beta) criteria [9], the 20 patients were diagnosed as having de novo CM during their first visit, i.e. they did not have a previous history of medication overuse. As a confirmation, we ensured that patients’ mean monthly tablet intake (2.8 ± 3.1 tablets/month; Table 1) was below the lower limit set by the International Classification Committee for medication overuse [9]. All patients had an established history of episodic migraine without aura (ICHD-3 beta code 1.1), and used nonsteroidal anti-inflammatory drugs as acute medication. With the exception of four patients who had mild headaches (mean visual analogue scale score = 2.5) without migrainous features, all of the patients with CM underwent the MRI scans during a headache-free state. Inclusion criteria were as follows: no history of other neurological diseases, systemic hypertension, diabetes or other metabolic disorders, connective or autoimmune diseases, medically treated depression, and/or any other type of primary or secondary headache. Patients did not always experience the headaches on the same side. To avoid the bias of pharmacologic treatment, no prophylactic treatments were allowed during the previous 3 months. For comparison, we enrolled 20 HCs of comparable age and sex distribution, who were recruited from among medical school students and healthcare professionals. The HCs had no personal or familial history (1st- or 2nd-degree relatives) of migraine or any detectable medical conditions and were not on any regular medications. The HCs were randomly scanned between patients. To avoid variability owing to hormonal changes, female participants underwent MRI outside of their pre-menstrual or menstrual periods. All scanning sessions were performed in the afternoon (16:00–19:00). For both HCs and patients, additional exclusion criteria were abnormal structural MR images of the brain and/or abnormal pathological findings, including WM lesions. All participants received a complete description of the study and granted written informed consent. The ethical review board of the Faculty of Medicine, University of Rome, Italy, approved the project.

**Table 1:** Demographic data from patients with chronic migraine (CM) and healthy controls (HCs) and the headache profile of the patients

	HCs (*n* = 20)	Patients with CM (*n* = 20)
Women (*n*)	13	14
Age (years)	28.5 ± 4.1	31.3 ± 10.2
Disease duration (years)		15.0 ± 13.1
Days with headache/month (*n*)		23.0 ± 6.8
Severity of headache attacks (0–10)		7.6 ± 1.6
Duration of the chronic headache (months)		17.1 ± 29.3
Tablet intake/month (*n*)		2.8 ± 3.1

Data are expressed as the mean ± the standard deviation

### Imaging protocols

A Siemens Magnetom Verio 3-Tesla scanner was used to acquire all images. Structural scans of the brain were acquired for each participant using a T1-weighted three-dimensional sagittal magnetisation-prepared rapid gradient echo sequence with the following parameters: 176 slices, repetition time = 1900 ms, echo time = 2.93 ms, slice thickness = 1 mm, and an in-plane resolution of 0.508 × 0.508 mm. The raw and preprocessed images were manually inspected for artefacts and image quality. Moreover, the ‘check sample homogeneity’ function in CAT12 (http://www.neuro.uni-jena.de) was used to identify images with poor quality and incorrect preprocessing. None of the acquired and preprocessed image series showed abnormalities.

### Data processing and analysis

Image data processing was conducted using SPM12 (www.fil.ion.ucl.ac.uk), and the CAT12 toolbox in the MatLab environment (www.mathworks.com) was used to perform the VBM analysis [10]. The images acquired for each participant were reoriented to have the same point of origin (anterior commissure) and spatial orientation. A non-linear deformation field was estimated that best overlaid the tissue probability maps on the individual subjects’ images. Three tissue components, namely the GM, WM, and cerebral spinal fluid (CSF), were obtained to calculate the overall tissue volume (GM, WM, and CSF volume) and total intracranial volume in the native space. Afterwards, all of the native-space tissue segments were registered to the standard Montreal Neurological Institute template (the standard included in SPM12) using the affine registration algorithm. The diffeomorphic anatomical registration through the exponentiated lie algebra (DARTEL) toolbox was applied to all participants’ GM and WM to refine the inter-subject registration. In the last step of DARTEL, the GM tissues are modulated using a non-linear deformation approach to compare the relative GM volume adjusted for individual brain size. Furthermore, the voxel values in the tissue maps are modulated by the Jacobian determinant that was calculated during spatial normalization [11]. Once the preprocessing pipeline was completed, a quality check was performed using a CAT12 toolbox to assess the homogeneity of the GM tissues. Lastly, each participant’s modulated and normalised GM tissue segments were smoothed with an 8-mm full width at half maximum Gaussian filter.

### Statistical analysis

We used CAT12 for all of the statistical analyses. First, a two-sample *t*-test was performed to compare the GM volume between patients and HCs. For all analyses, we included age, sex, and total intracranial volume as covariates (Additional file 1: Figure S1). The patients’ relative GM volume changes were initially assessed at a threshold of *p* < 0.05 (corrected for multiple comparisons [family-wise error]). However, the small sample size and previous VBM evidence showing the involvement of specific brain structures in the process of migraine recurrence [8, 12] prompted us to also perform an exploratory analysis using less-conservative uncorrected thresholds of *p* < 0.001 and *p* < 0.005 throughout the whole brain. Thereafter, to identify whether the regional GM volume changes were correlated with patients’ clinical features, we performed multiple univariate regression analyses using the CAT12 model design tool that included the severity of the headache attacks (0–10), disease duration (years), number of days per month with headaches (n), attack duration (h), number of tablets taken per month (n), and duration of the chronic headache phase (months) as independent variables. These inferences have been performed at a level of *p* < 0.001 uncorrected.

## Results

All participants completed the study. The demographic data and clinical profiles of the patients are shown in Table 1. The patients and HCs were not different in terms of their age or sex distributions.

**Fig. 1:**
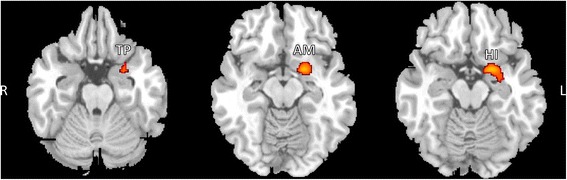
SPM regions superimposed on a high-resolution T1-weighted scan show decreased GM volume in patients with CM compared to HCs (*p* < 0.001 uncorrected). Areas with significantly reduced GM volume are observed in the left amygdala (AM), left temporal pole (TP), and left hippocampus (HI). L = left, R = right

**Fig. 2:**
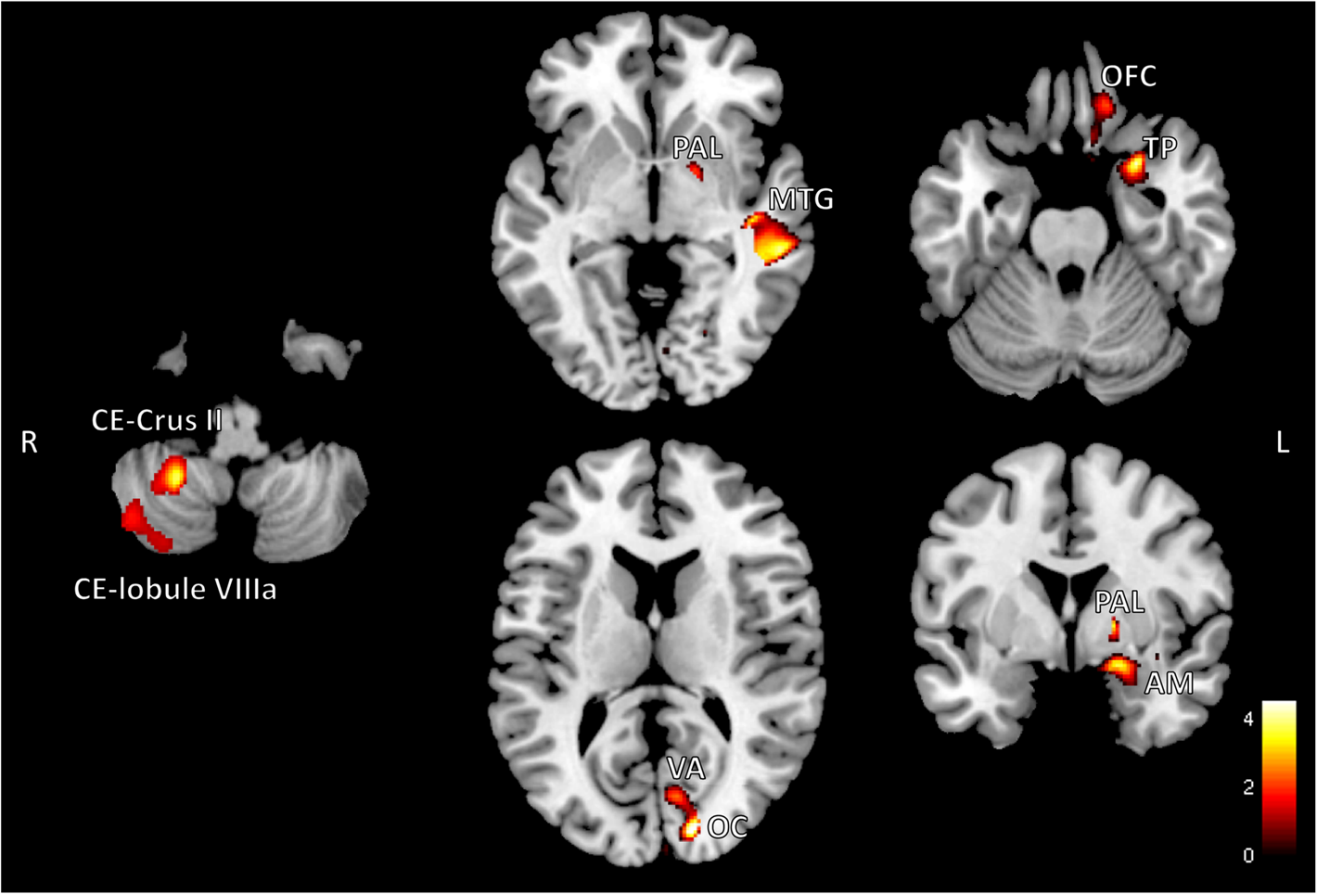
SPM regions superimposed on a high-resolution T1-weighted scan show decreased GM volume in patients with CM compared to HCs (*p* < 0.005 uncorrected). Areas with significantly reduced GM volume are observed in the cerebellum (CE), left primary occipital cortex (OC), visual association area (VA), left middle temporal gyrus (MTG), left amygdala (AM), left temporal pole (TP), left pallidum (PAL), and left orbitofrontal cortex (OFC). Labels of the cerebellum stem from the Spatially Unbiased Infraorbital Template atlas. L = left, R = right

Regarding GM differences, the total cerebral GM volume was significantly lower in patients with CM than it was in HCs (617 ± 62.6 mL vs. 658.1 ± 62.4 mL: *t* = 2.055, *p* = 0.047). In the analysis corrected for multiple comparisons (*p* < 0.05 family-wise error corrected), we did not detect any regions with significant GM changes between HCs and patients with CM. Therefore, to further explore the GM volume changes in patients with CM in comparison with HCs, we assessed the results at more-liberal thresholds of *p* < 0.001 and *p* < 0.005 (uncorrected). At p < 0.001, we found one cluster of regions that showed significant GM volume reductions in patients with CM in comparison with HCs, including the left amygdala, left temporal pole, and left hippocampus (Fig. 1 and Table 2). At p < 0.005, we noted that patients with CM showed GM volume reductions in the right cerebellum (lobule VIIIa and Crus II, as - as defined in the Spatially Unbiased Infraorbital Template atlas [13]), left middle temporal gyrus (MTG), left amygdala, left temporal pole, left pallidum, left orbitofrontal cortex (OFC), left primary occipital cortex (Brodmann area [BA] 17), and visual association area (BA18) compared to HCs (Fig. 2 and Table 3). No significant increases in GM volume were found in patients with CM compared with HCs.

**Table 2:** Clusters of significant gray matter reduction in patients with chronic migraine vs. healthy controls using uncorrected maps at *p* < 0.001

Anatomical regions	Brodmann area	Cluster extent (mm^3^)	Montreal Neurological Institute coordinates (x, y, z)	Peak Z Score	T value	*P* value (cluster level p uncorrected)
*Cluster 1*		390				0.0224
L Amygdala			−20, 3, −15	4.34	5.04	
L Temporal pole			−26, 9, −26	3.23	3.52	
L Hippocampus			−31, −5, −15	3.20	3.47	

*R* right, *L* left

**Table 3:** Clusters of significant gray matter reduction in patients with chronic migraine vs. healthy controls using uncorrected maps at *p* < 0.005

Anatomical regions	Brodmann area	Cluster extent (mm^3^)	Montreal Neurological Institute coordinates (x, y, z)	Peak Z Score	T value	*P* value (cluster level p uncorrected)
*Cluster 1*		1624				0.0085
R Cerebellum (lobule VIIIa)			27, −48, −48	4.01	4.53	
R Cerebellum (Crus II)			45, −64, −48	3.01	3.22	
*Cluster 2*		1707				0.0073
L Middle temporal gyrus	BA21		−58, −32, 0	3.75	4.17	
*Cluster 3*		1620				0.009
L Amygdala			−20, 2, −19	3.67	4.06	
L Temporal pole	BA38		−27, 7, −25	3.62	4.0	
L Pallidum			−18, 0, 3	3.48	3.81	
L Orbitofrontal cortex	BA11		−10, 30, −18	2.73	2.94	
*Cluster 4*		582				0.037
L Primary occipital cortex	BA17		−9, −74, 10	3.83	3.25	
L visual association area	BA18		−14, −90, 16	3.64	3.5	

*R* right, *L* left

The univariate regression analysis showed that the lower the volume of the cerebellum (bilaterally), the longer the disease duration and the lower the monthly tablet intake in patients with CM (Table 4).

**Table 4:** VBM results of correlation analysis on GM morphometry with CM patients’ clinical features at significance level of *p* < 0.001 (uncorrected) and adjusted for age, gender and total intracranial volume

Anatomical regions	Cluster extent (mm^3^)	Montreal Neurological Institute coordinates (x, y, z)	T value	*P* value (cluster level p uncorrected)	Clinical feature
	711			0.0014	Tablet intake/month (n)
R Cerebellum (lobule VIIIa)		39, −41, −48	5.71		
R Cerebellum (Crus II)		50, −48, −47	5.05		
	685			0.0017	
L Cerebellum (lobule VI)		−17, −68, −17	6.03		
L Cerebellum (Crus I)		−15, −78, −21	4.33		
	625			0.028	Disease duration (years)
R Cerebellum (Crus II)		24, −68, −39	4.95		

*R* right, *L* left

## Discussion

The present VBM study failed to find significant GM changes in de novo patients diagnosed with CM in comparison with HCs. Thus, at first glance, the present results do not support earlier whole-brain VBM studies that revealed gross significant abnormal GM volume changes in areas ascribable to the processing of pain [2–6] and multisensory integration [5] in patients with CM versus healthy individuals. However, as mentioned above, many of these studies had major sources of bias, which may have affected their results. Here, we have avoided such biases by excluding patients with a previous or actual history of MOH, concomitant use of preventive medications, patients with multiple headache diagnosis, and with structural WM abnormalities [4]. Our results thus may be more representative of the actual GM volume in patients with CM.

Considering the small sample size of the present study and the fact that migraine, even in its chronic form, is a functional disorder of the brain where morphological abnormalities, if present, might manifest as subtle regional dysfunctions, we explored the imaging data using more-liberal uncorrected thresholds of *p* < 0.001 and *p* < 0.005. At a threshold of p < 0.001, patients with CM displayed less GM volume in the left amygdala, left temporal pole, and left hippocampus compared to HCs. Furthermore, by lowering the threshold to p < 0.005 uncorrected, we identified four clusters of regions that showed GM volume reductions in patients with CM compared to HCs, including the cerebellum; left occipital areas (BA17/BA18); left MTG; and left temporal pole, amygdala, pallidum, and OFC. Brain structures within these clusters have previously been linked to pain processing, and the structure and/or function of several of these regions is known to be abnormal in patients with episodic and chronic migraine with or without medication overuse. Below, we discuss the importance of each of these clusters in turn, as well as our finding that the clinical features of CM, including the disease duration and tablet intake per month, were associated with patients’ morphometric data.

### Subtle GM volume changes in the left temporal pole, amygdala, hippocampus, pallidum, and OFC

The so-called mesocorticolimbic reward circuit consists of a complex network of cortical and subcortical regions that are responsible for the effects of positive and negative reinforcement (reward and aversion) [14]. Together, the regions in this network, including the OFC, pallidum, amygdala, hippocampus, and temporal pole, integrate information related to reward processing, emotion, and memory to modulate striatal activity [15]. In brief, dense OFC fibres converge in the central and lateral parts of the ventral striatum (activated by reward-related behavioural paradigms). The amygdala and temporal pole play key roles in the emotional coding and recalling of salient stimuli [16]. The amygdala also projects to the ventral striatum, which in turn sends efferent projections to the pallidum [17]. The reward circuit is a critical component of the brain disease model of addiction [18], of which CM due to medication overuse is thought to belong [19]. In particular, volume reductions in the amygdala and hippocampus have been previously reported in adults affected by substance abuse disorders and in their apparently healthy offspring, indicating a possible neurodevelopmental component [14, 18]. Other studies have demonstrated that the temporal pole [20], amygdala [3, 6], and pallidum [21] are involved, with a variable level of statistical significance, in the process of migraine chronification. In interictal CM, the amygdala was previously found to be atypically connected with regions in the superior frontal cortex and occipital cortex [22]. However, to the best of our knowledge, no other study has specifically reported a true reduction of GM volume in the OFC of patients with CM without a history of medication overuse. Neuroimaging studies of patients with CM with a history of medication overuse identified abnormal blood-oxygen-level dependent activity [23], GM volume [5, 12, 24], and metabolism [25] in the OFC region. In a VBM study, Riederer et al. [24] observed that patients with MOH had significantly less GM in the OFC and that the reduced GM volume in this area was associated with the treatment response. In the ^18^F-fluorodeoxyglucose positron emission tomography study by Fumal et al. [25], patients with MOH who underwent successful withdrawal from acute medications had greater metabolism reductions in the OFC after withdrawal than they did before withdrawal, leading the authors to conclude that the latter could predispose a subgroup of migraineurs to overuse analgesics. Interestingly, in episodic migraineurs, new-onset medication overuse was associated with baseline poor performance in tasks related to orbitofrontal function [26]. Considering these findings, we propose that patients with slightly reduced GM volume in regions that are part of the mesocorticolimbic reward circuit may be at risk of decreasing the threshold for the tendency to consume analgesics.

### Subtle GM volume changes in the cerebellum

In animals and humans, the deep cerebellar nuclei process noxious stimuli [27–29] and participate in pain perception and inhibition through their connections with the brainstem nuclei and thalamus [30, 31]. In HCs, when the cerebellar activity is forcibly enhanced, e.g. through neuromodulatory techniques, then the pain threshold is increased, i.e. the antinociceptive effects of the cerebellum are heightened [32]. Here, we found that the GM volume within cerebellar lobule VIIIa and Crus II was slightly reduced in patients with CM compared to HCs. Cerebellar lobule VIIIa, which represents part of the face within the cerebellum [33], has been shown to play a role in trigeminal nociception [31], while Crus II seems to be more active during non-noxious emotional processing [29, 34] and cognitive associative learning [35]. Our exploratory volumetric MRI data partially agree with those of Bilgiç and co-workers [4] who found reduced right, as well as left, cerebellum volume in patients with CM with a high rate of medication overuse and under migraine prophylaxis in comparison with HCs. Interestingly, previous neuroimaging studies of patients with CM with excessive acute medication intake identified elevations in the cerebellar metabolism [25] and volume [12]. We think that these observations are still in line with our exploratory results, as our correlation analysis revealed that higher acute medication intake was linked to higher cerebellar GM volume. Overall, we reason that abnormal macrostructural patterns in the cerebellum may be a predisposing factor that may lead to MOH development. Moreover, we found that the longer the history of migraine, the lower was the neuronal volume of the cerebellum, an observation that could be interpreted as indicating that time-dependent plastic changes in the cerebral microstructure are correlated with the chronic perpetuation of migraine attacks, or that the cerebellum is anatomically susceptible to the emotional/cognitive consequences of a chronic disorder. Our findings in patients with CM coincide with those showing that morpho-functional abnormalities in the periaqueductal gray area, which is interconnected with the cerebellum [36], are positively correlated with the disease duration in patients with CM [37], i.e. the longer the disease duration the higher the dysfunctional cerebellar antinociceptive effects.

### Subtle GM volume changes in the MTG

Located on the lateral surface of the temporal pole, the MTG is involved in several cognitive functions. Reduced GM volume and changes in the functional connectivity of the left MTG have been linked to the severity of the clinical symptoms associated with social anxiety or phobia, of which a core feature is anticipatory anxiety, i.e. a state of continuous alertness for an imminent or likely threatening event [38], such as a headache. It was postulated that subjects affected by anticipatory anxiety are more prone to engage in avoidant behaviours from potential threatening events [39]. Social phobia is a disorder that is frequently diagnosed in individuals with juvenile [40] and adult [39, 41] CM, and, very likely, even in animal models of CM [42]. Some researchers have suggested that being affected by both CM and social phobia configures a state of phobic avoidance that is associated with the fear of a migraine attack, which may explain why some patients take analgesics at the smallest indication of a headache and why such patients may be at risk of developing medication overuse or of decreasing the threshold for analgesic consumption [39, 43]. However, since we did not assess patients’ psychiatric profiles, and considering the exploratory nature of our uncorrected findings, we cannot draw a definitive conclusion about the link between reduced GM volume in the MTG and social phobia in terms of its ability to promote migraine chronification and medication intake. To clarify this, future studies investigating the cerebral microstructure and connectivity in patients with CM with/without medication overuse should include assessments of patients’ psychiatric profiles.

### Subtle GM volume changes in the occipital areas

In the present study, we discovered that patients with CM had slightly reduced GM volume in visual area 17 and visual association area 18 compared to HCs. It is worth noting that pain can be related to vision. Studies of cortical function show that tonic pain induces marked spontaneous [44] or evoked [45] electroencephalographic and functional imaging [46] changes in the occipital regions. Recently, in a group of patients with episodic migraines, we found reductions in the functional connectivity between the visuo-spatial system and the so-called default mode network between attacks [47], while during attacks, the connectivity was reduced between the executive and dorso-ventral visual attention networks [48], stressing that occipital areas could be involved in the attentional processes to pain and in some aspects of pain representation [49]. Notably, the visual presentation of affective pictures modulates occipital functional activation and, at the same time, pain perception differently in patients with CM than it does in HCs [50], perhaps through direct occipital-to-brainstem trigeminal nuclei connections [51]. Therefore, our results in patients with CM tend to show the morphological correlates of aberrant attentional processes to head pain and of anomalous representations of pain.

### Limitations

Certain limitations of the present study should be acknowledged. First, the sample size was small, thus GM volume changes in patients versus HCs were apparent only when data was assessed with very liberal uncorrected thresholds. Additionally, we did not analyse the psychiatric profiles of the patients, although we think that patients’ social anxiety symptomology may contribute to their clinico-morphological status.

## Conclusions

In summary, our study did not find significant differences in GM volume between CM patients and HCs. However, using more-liberal thresholds, we noted that patients with CM showed reduced GM volume in the MTG and OFC, which are known to be involved in avoidant and addictive behaviours, respectively. Based on these findings and the results of our correlation analysis, we speculate that these abnormalities could lead, at least in a subgroup of patients, to the development and continuation of maladaptive acute medication usage. Although these exploratory findings should be interpreted with caution, they provide a basis for performing future investigations in CM using more-sophisticated MRI techniques.

## Additional file

Additional file 1: Figure S1. Results of the SPM analysis comparing chronic migraine patients and healthy controls. The design matrix (right) and statistically significant clusters are shown on a glass brain in the three orthogonal planes (left),) with the results shown at a threshold of *p* < 0.05 (corrected for multiple comparisons) [A], *p* < 0.001 uncorrected [B], and *p* < 0.005 uncorrected [C]. (TIFF 1575 kb)

## Abbreviations

BA: Brodmann area
CM: Chronic migraine
CSF: Cerebral spinal fluid
DARTEL: Diffeomorphic anatomical registration through the exponentiated lie algebra
GM: Gray matter
HC: Healthy control
ICHD-3 beta: International Classification of Headache Disorders, 3rd edition, beta
MOH: Medication overuse headache
MRI: Magnetic resonance imaging
MTG: Middle temporal gyrus
OFC: Orbitofrontal cortex
VBM: Voxel-based morphometry
WM: White matter.

## References

[1] Wang SJ, Fuh JL, Lu SR (2000). Chronic daily headache in Chinese elderly: prevalence, risk factors, and biannual follow-up. Neurology.

[2] Schmidt-Wilcke T, Gänssbauer S, Neuner T (2008). Subtle grey matter changes between migraine patients and healthy controls. Cephalalgia.

[3] Valfrè W, Rainero I, Bergui M, Pinessi L (2008). Voxel-based morphometry reveals gray matter abnormalities in migraine. Headache.

[4] Bilgic B, Kocaman G, Arslan AB (2016). Volumetric differences suggest involvement of cerebellum and brainstem in chronic migraine. Cephalalgia.

[5] Lai T-H, Chou K-H, Fuh J-L (2016). Gray matter changes related to medication overuse in patients with chronic migraine. Cephalalgia.

[6] Neeb L, Bastian K, Villringer K (2017). Structural gray matter alterations in chronic migraine: implications for a progressive disease?. Headache.

[7] Whitwell JL (2009). Voxel-based morphometry: an automated technique for assessing structural changes in the brain. J Neurosci.

[8] Coppola G, Di Renzo A, Tinelli E (2015). Evidence for brain morphometric changes during the migraine cycle: a magnetic resonance-based morphometry study. Cephalalgia.

[9] Headache Classification Committee of the International Headache Society (IHS) (2013) The International Classification of Headache Disorders, 3rd edition (beta version). Cephalalgia 33:629–808 10.1177/0333102413485658 23771276

[10] Ashburner J, Friston KJ (2000). Voxel-based morphometry--the methods. NeuroImage.

[11] Good CD, Johnsrude IS, Ashburner J (2001). A Voxel-based Morphometric study of ageing in 465 normal adult human brains. NeuroImage.

[12] Riederer F, Marti M, Luechinger R (2012). Grey matter changes associated with medication-overuse headache: correlations with disease related disability and anxiety. World J Biol Psychiatry.

[13] Diedrichsen J (2006). A spatially unbiased atlas template of the human cerebellum. NeuroImage.

[14] Makris N, Oscar-Berman M, Jaffin SK (2008). Decreased volume of the brain reward system in alcoholism. Biol Psychiatry.

[15] Sawyer KS, Oscar-Berman M, Barthelemy OJ (2017). Gender dimorphism of brain reward system volumes in alcoholism. Psychiatry Res Neuroimaging.

[16] Hortensius R, Terburg D, Morgan B et al (2017) The Basolateral Amygdalae and Frontotemporal network functions for threat perception. ENEURO 4(1) 10.1523/ENEURO.0314-16.2016 PMC5368167 28374005

[17] Dreher J-C, Trembaly L (2016) Decision neuroscience an integrative perspective. Academic Press, Amsterdam

[18] Volkow ND, Koob GF, McLellan AT (2016). Neurobiologic advances from the brain disease model of addiction. N Engl J Med.

[19] Calabresi P, Cupini LM (2005). Medication-overuse headache: similarities with drug addiction. Trends Pharmacol Sci.

[20] Schwedt TJ, Chong CD, Wu T (2015). Accurate classification of chronic migraine via brain magnetic resonance imaging. Headache J Head Face Pain.

[21] Maleki N, Becerra L, Nutile L (2011). Migraine attacks the basal ganglia. Mol Pain.

[22] Schwedt TJ, Schlaggar BL, Mar S (2013). Atypical resting-state functional connectivity of affective pain regions in chronic migraine. Headache.

[23] Ferraro S, Grazzi L, Muffatti R (2012). In medication-overuse headache, FMRI shows long-lasting dysfunction in midbrain areas. Headache.

[24] Riederer F, Gantenbein AR, Marti M (2013). Decrease of gray matter volume in the midbrain is associated with treatment response in medication-overuse headache: possible influence of orbitofrontal cortex. J Neurosci.

[25] Fumal A, Laureys S, Di Clemente L (2006). Orbitofrontal cortex involvement in chronic analgesic-overuse headache evolving from episodic migraine. Brain.

[26] Gómez-Beldarrain M, Carrasco M, Bilbao A, García-Moncó JC (2011). Orbitofrontal dysfunction predicts poor prognosis in chronic migraine with medication overuse. J Headache Pain.

[27] Siegel P, Wepsic JG (1974). Alteration of nociception by stimulation of cerebellar structures in the monkey. Physiol Behav.

[28] Moulton EA, Schmahmann JD, Becerra L, Borsook D (2010). The cerebellum and pain: passive integrator or active participator?. Brain Res Rev.

[29] Moulton EA, Elman I, Pendse G (2011). Aversion-related circuitry in the cerebellum: responses to noxious heat and unpleasant images. J Neurosci.

[30] Saab CY, Willis WD (2002). Cerebellar stimulation modulates the intensity of a visceral nociceptive reflex in the rat. Exp Brain Res.

[31] Mehnert J, Schulte L, Timmann D, May A (2017). Activity and connectivity of the cerebellum in trigeminal nociception. NeuroImage.

[32] Pereira M, Rafiq B, Chowdhury E (2017). Anodal cerebellar tDCS modulates lower extremity pain perception. NeuroRehabilitation.

[33] Manni E, Petrosini L (2004). A century of cerebellar somatotopy: a debated representation. Nat Rev Neurosci.

[34] Helmchen C, Mohr C, Erdmann C (2003). Differential cerebellar activation related to perceived pain intensity during noxious thermal stimulation in humans: a functional magnetic resonance imaging study. Neurosci Lett.

[35] Timmann D, Drepper J, Frings M (2010). The human cerebellum contributes to motor, emotional and cognitive associative learning. A review Cortex.

[36] Teune TM, van der Burg J, van der Moer J, et al (2000) Topography of cerebellar nuclear projections to the brain stem in the rat. In: Prog. Brain Res. pp 141–172 10.1016/S0079-6123(00)24014-4 10943123

[37] Welch KMA, Nagesh V, Aurora SK, Gelman N (2001). Periaqueductal gray matter dysfunction in migraine: cause or the burden of illness?. Headache.

[38] Yun J-Y, Kim J-C, Ku J (2017). The left middle temporal gyrus in the middle of an impaired social-affective communication network in social anxiety disorder. J Affect Disord.

[39] Corchs F, Mercante JPP, Guendler VZ (2006). Phobias, other psychiatric comorbidities and chronic migraine. Arq Neuropsiquiatr.

[40] Masruha MR, Lin J, Minett TSC (2012). Social anxiety score is high in adolescents with chronic migraine. Pediatr Int.

[41] Serafini G, Pompili M, Innamorati M (2012). White matter hyperintensities and self-reported depression in a sample of patients with chronic headache. J Headache Pain.

[42] Zhang M, Liu Y, Zhao M (2017). Depression and anxiety behaviour in a rat model of chronic migraine. J Headache Pain.

[43] Peres MFP, Mercante JPP, Guendler VZ (2007). Cephalalgiaphobia: a possible specific phobia of illness. J Headache Pain.

[44] Backonja M, Howland EW, Wang J (1991). Tonic changes in alpha power during immersion of the hand in cold water. Electroencephalogr Clin Neurophysiol.

[45] Coppola G, Serrao M, Currà A (2010). Tonic pain abolishes cortical habituation of visual evoked potentials in healthy subjects. J Pain.

[46] Peyron R, Laurent B, García-Larrea L (2000). Functional imaging of brain responses to pain. A review and meta-analysis (2000). Neurophysiol Clin.

[47] Coppola G, Di Renzo A, Tinelli E (2016). Thalamo-cortical network activity between migraine attacks: insights from MRI-based microstructural and functional resting-state network correlation analysis. J Headache Pain.

[48] Coppola G, Di Renzo A, Tinelli E (2016). Thalamo-cortical network activity during spontaneous migraine attacks. Neurology.

[49] Peyron R, García-Larrea L, Grégoire MC et al (1999) Haemodynamic brain responses to acute pain in humans: sensory and attentional networks. Brain:1765–1780 10.1093/brain/122.9.1765 10468515

[50] de Tommaso M, Ricci K, Laneve L (2013). Virtual visual effect of hospital waiting room on pain modulation in healthy subjects and patients with chronic migraine. Pain Res Treat.

[51] Sava SL, de Pasqua V, Magis D et al (2014) Effects of visual cortex activation on the Nociceptive blink reflex in healthy subjects. PLoS One 9:e100198 10.1371/journal.pone.0100198 PMC4061134 24936654

