# Supplementary figures and images for: Cerebral gray matter volume in patients with chronic migraine: correlations with clinical features
Source: J Headache Pain. 2017 Dec 8;18(1):115. doi: 10.1186/s10194-017-0825-z (PMC5762618; doi:10.1186/s10194-017-0825-z)

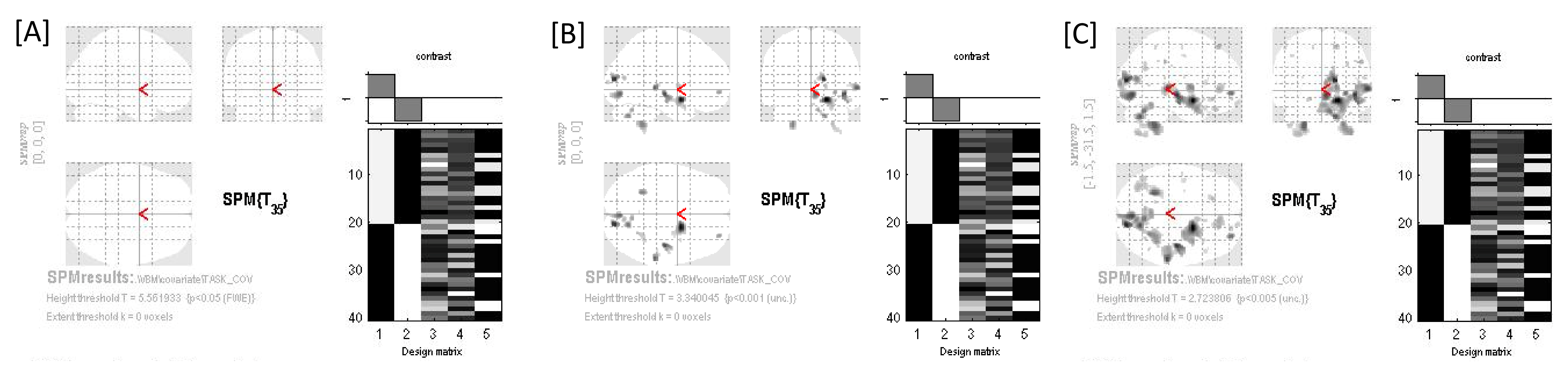

Supplement: Additional file 1: Figure S1. — Results of the SPM analysis comparing chronic migraine patients and healthy controls. The design matrix (right) and statistically significant clusters are shown on a glass brain in the three orthogonal planes (left),) with the results shown at a threshold of p < 0.05 (corrected for multiple comparisons) [A], p < 0.001 uncorrected [B], and p < 0.005 uncorrected [C]. (TIFF 1575 kb) [file 10194_2017_825_MOESM1_ESM.tif]
